# Supplementary material for: Macrophage Polarization Related to Biomimetic Calcium Phosphate Coatings: A Preliminary Study
Source: Materials (Basel). 2022 Dec 29;16(1):332. doi: 10.3390/ma16010332 (PMC9822186; doi:10.3390/ma16010332)
Supplement: Supplementary file 1 [file materials-16-00332-s001.zip › materials-1912632-supplementary.pdf]

Supplementary Material

Table S1. Primer sequences used for reverse transcription-quantitative polymerase reaction (RT-qPCR).

Gene	5'-3'	Sequence
TNF-α	Forward	CCCTCACACTCAGATCATCTTCT
	Reverse	GCTACGACGTGGGCTACAG
Arg1	Forward	CTCCAAGCCAAAGTCCTTAGAG
	Reverse	AGGAGCTGTCATTAGGGACATC
CD206	Forward	CTCTGTTCAGCTATTGGACGC
	Reverse	CGGAATTTCTGGGATTCAGCTTC
GAPDH	Forward	AGGTCGGTGTGAACGGATTTG
	Reverse	TGTAGACCATGTAGTTGAGGTCA
